# Successes and Challenges of Implementing Tobacco Dependency Treatment in Health Care Institutions in England

**DOI:** 10.3390/curroncol29050299

**Published:** 2022-05-20

**Authors:** Sanjay Agrawal, Zaheer Mangera, Rachael L. Murray, Freya Howle, Matthew Evison

**Affiliations:** 1 Institute for Lung Health, Department of Respiratory Medicine, Glenfield Hospital, Groby Road, Leicester LE3 9QP, UK; sanjay.agrawal@uhl-tr.nhs.uk; 2 Department of Respiratory Medicine, North Middlesex University Hospital, Sterling Way, London N18 1QX, UK; zaheer.mangera@nhs.net; 3 Academic Unit of Lifespan and Population Health, School of Medicine, Nottingham University, Nottingham NG7 2RD, UK; rachael.murray@nottingham.ac.uk; 4 Greater Manchester CURE Programme Team, Greater Manchester Cancer Alliance, The Christie NHS Foundation Trust, Wilmslow Road, Manchester M20 4BX, UK; f.howle@nhs.net; 5 Lung Cancer & Thoracic Surgery Directorate, Wythenshawe Hospital, Manchester University NHS Foundation Trust, Manchester, Southmoor Road, Manchester M23 9LT, UK

**Keywords:** NHS long term plan, tobacco dependency treatment

## Abstract

There is a significant body of evidence that delivering tobacco dependency treatment within acute care hospitals can deliver high rates of tobacco abstinence and substantial benefits for both patients and the healthcare system. This evidence has driven a renewed investment in the UK healthcare service to ensure all patients admitted to hospital are provided with evidence-based interventions during admission and after discharge. An early-implementer of this new wave of hospital-based tobacco dependency treatment services is “the CURE project” in Greater Manchester, a region in the North West of England. The CURE project strives to change the culture of a hospital system, to medicalise tobacco dependency and empower front-line hospital staff to deliver an admission bundle of care, including identification of patients that smoke, provision of very brief advice (VBA), protocolised prescription of pharmacotherapy, and opt-out referral to the specialist CURE practitioners. This specialist team provides expert treatment and behaviour change support during the hospital admission and can agree a support package after discharge, with either hospital-led or community-led follow-up. The programme has shown exceptional clinical effectiveness, with 22% of all smokers admitted to hospital abstinent from tobacco at 12 weeks, and exceptional cost-effectiveness with a public value return on investment ratio of GBP 30.49 per GBP 1 invested and a cost per QALY of GBP 487. There have been many challenges in implementing this service, underpinned by the system-wide culture change and ensuring the good communication and engagement of all stakeholders across the complex networks of the tobacco control and healthcare system. The delivery of hospital-based tobacco dependency services across all NHS acute care hospitals represents a substantial step forward in the fight against the tobacco epidemic.

## 1. Introduction

Smoking tobacco is unique in the level of harm and destruction it causes. It is both uniquely addictive and uniquely poisonous, a deadly combination that has led to the death of more than 8 million people in the United Kingdom (UK) alone in the last 50 years of this tobacco epidemic [1]. With the uptake of smoking declining to the lowest recorded levels, the most imperative public health intervention is to support those that currently smoke to stop. There is an extensive evidence-base, ranging from randomised controlled trials to meta-analyses to Cochrane reviews, demonstrating highly effective interventions and treatments for tobacco dependency [2,3,4,5,6,7], but the greatest challenge for healthcare systems is maximising the access and uptake to these interventions for all people that smoke. This article will present the history of funding models for stop smoking services in the UK, including the tragic decline in funding and uptake in the last decade but also a new hope, with a significant new investment within the National Health Service (NHS) that focuses on the treatment of tobacco dependency in patients admitted to acute care hospitals. The successes and challenges of a vanguard service implementing this service on a regional scale are also presented and discussed.

## 2. The History of Tobacco Control and the National Health Service in England

**Figure 1:**
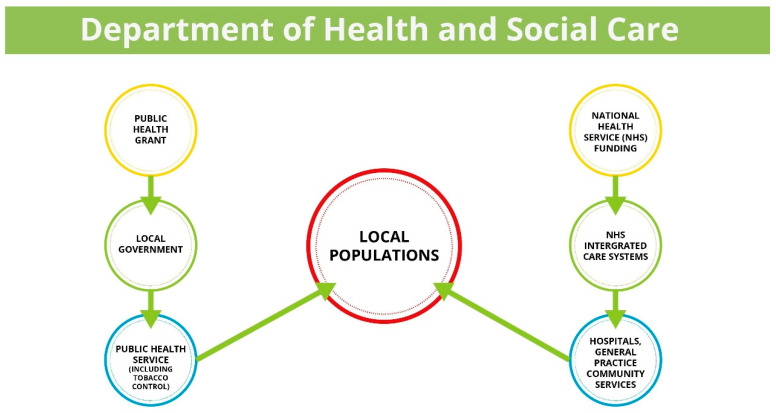
Funding flow from the Department of Health and Social Care to the NHS and Local Government in England.

**Figure 2:**
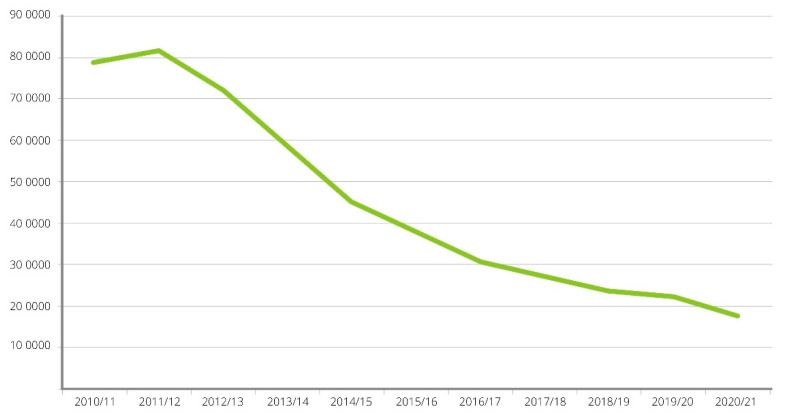
Persons setting a quit date using NHS stop smoking services 2010/11 to 2020/21.

The NHS, introduced in 1948, provides healthcare that is universal, comprehensive, and free at the point of delivery, funded by general taxation [8]. Each of the four nations of the UK determine their own NHS structure, health policy, priorities, and the degree of integration between health and social care. Treatment for tobacco dependency was recognised as a major pillar of tobacco control by the Royal College of Physicians (RCP) in the middle of the last century [9], and despite the availability of medicinal nicotine replacement therapy in the 1980s, it was not until the UK government white paper “Smoking Kills” was published in 1998 that “Stop Smoking Services” were funded and set up across England [10]. These new NHS Stop Smoking Services used evidence-based guidelines [11] to provide a combination of behavioural support and pharmacotherapy to treat tobacco dependence. Smoking cessation practitioners were trained utilising the newly commissioned National Centre for Smoking Cessation and Training (NCSCT), which also provided resources on treatment models that were applicable to both NHS and community settings [12]. The National Institute of Health and Care Excellence (NICE), the body tasked with producing evidence-based guidelines and quality standards, and designed to improve outcomes for patients accessing NHS care [13], produced several smoking-related clinical guidelines [14,15,16,17,18,19] that supported the application of evidence-based treatment and commissioning decisions to fund NHS Stop Smoking Services. However, the responsibility for public health, including tobacco dependency treatment services, was transferred from the NHS in England to local government in 2013 [20]. Public health services provided by local government are funded by the Department of Health and Social Care (DHSC) through the “Public Health Grant” (Figure 1) [21]. Between 2015–2016 and 2020–2021 NHS budgets have grown by 3% per capita, in contrast to cuts in the Public Health Grant of 24%, with a disproportionate reduction in tobacco control spending by local government during this period [22]. At their peak in 2011/2012 NHS Stop Smoking Service treated 816,444 people; however, use of these services dropped to 178,815 in 2020/2021 [23] (Figure 2). The explanation for the reduction in use of these services is multifactorial and may include funding cuts to local government stop smoking services [22], reduction in national mass media promotional campaigns [24], disjointed referral and treatment pathways between NHS and local government services [25], the disinvestment in regional and subregional tobacco control alliances [26], and the use of e-cigarettes to support self-motivated quit attempts [27].

## 3. A New National Strategy for Tobacco Dependency Treatment in the NHS

The RCP has, once again, been a driving force in tobacco control, seeking to address the tragic reduction in funding and access to tobacco dependency treatment. In 2018, on its 500th anniversary, the RCP published “Hiding in plain sight; treating tobacco dependency in the NHS” [28], which started to change the language in tobacco control, to medicalise the disease of tobacco dependency and place the responsibility for the identification and treatment of this disease back within the NHS and on all healthcare professionals. Furthermore, NICE, in its latest update in relation to tobacco dependency, makes clear that the obligation is on health care professionals to identify the smoking status of their patients and lay out a wide range of evidence-based stop-smoking interventions that include NRT (including vaping) and other pharmacotherapy and behavioural support [19]. Whilst the guidance clearly outlines how smokers are best supported, it does not resolve the issues surrounding the funding of such interventions and services. A watershed moment for NHS tobacco dependency treatment in the UK is the NHS England Long Term Plan [29] that, amongst its many concerns, recognises that smokers are more likely to access healthcare, linked to half a million hospital admissions each year [30] and a key indicator of health inequality. The NHS long term plan, therefore, committed to ensuring that all patients admitted to hospital who smoke will be offered NHS-funded tobacco dependency interventions by 2023/2024. Future stages of the plan aim to include tobacco dependency interventions in maternity, mental health services, and high-risk outpatient settings [29]. Although the initial stages of this plan have been hampered by the COVID-19 pandemic [31], an initial pilot phase is underway involving early implementer sites across England. In addition, Integrated Care Systems (ICSs) are actively in the process of engaging with their partner hospitals and distributing funding depending on the local landscape, to invest in tobacco dependency advisors (TDA), project managers, and pharmacotherapy. The funding will continue into 2023–2024 with hospitals expected to report back on patient level data to NHS England. As this is a new horizon for many hospitals, given only 37% of hospitals had a stop smoking service in 2019 [32], the British Thoracic Society (BTS) have been commissioned by NHS England to provide a platform from which resources relating to the implementation of stop smoking services can be disseminated via web-based resources and webinars [33]. However significant challenges remain, and the lessons learned from the early-implementer sites demonstrate the need for (1) dedicated project management to launch and maintain services, (2) strong clinical leadership to drive through change (3) robust information and clinical systems to help identify and refer smokers, and (4) extensive training of patient-facing staff, including how to offer support and prescribing NRT. This also includes training of TDAs, which will require a substantial expansion, in order to meet the goals of the Long-Term Plan. Finally, ensuring the smooth transition of patients from hospital to community services after discharge is a further area of vulnerability, given the historic underinvestment in community services by local government.

## 4. Evidence-Base for Tobacco Dependency Treatment in the Acute Hospital Setting

The Ottawa model for smoking cessation (OMSC) was first implemented in 2006 at the University of Ottawa Heart Institute in Canada, to support the consistent and effective identification and treatment of tobacco users in secondary care settings. Implementing the OMSC involved a six-phase workplan, which includes a (1) baseline assessment, (2) development of a clinical tobacco treatment protocol, (3) adaptation of clinical management systems and tools, (4) staff training, (5) programme launch, and (6) ongoing quality improvement and education [34]. The model uses an application of the five A’s model: ask, advise, assess, assist and arrange, and is based around the core activities of training clinical staff to provide smoking cessation advice and support, recording of smoking status on patient charts, delivery of non-judgmental advice to quit to all people who smoke, inclusion of cessation counselling on patient care maps, bedside counselling delivered by trained nurse counsellors, timely provision of nicotine replacement therapy (NRT), automated telephone follow up, and referral to outpatient cessation sources [35]. Patients are contacted between 3 and 180 days following discharge via an automated telephone system, which assesses current smoking status, confidence in remaining smoke free, and use of cessation support; any patient indicating that they are currently smoking or who has low confidence in remaining smoke free are contacted by a stop smoking specialist. An evaluation of nine hospitals that first implemented the OMSC found that it increased delivery of stop smoking support and significantly increased long-term cessation rates [34]. Despite the reported attitudinal, managerial, and environmental challenges to its implementation, the OMSC was successfully implemented into routine practice across a variety of hospital settings. Furthermore, an evaluation of the OMSC in 14 Ontario hospitals showed significantly lower rates of all-cause readmissions, smoking related readmissions, and all cause emergency department visits at 30 days, 1 year, and 2 years after initial hospitalisation; furthermore, there was a significant decrease in mortality at 1 and 2 years [36]. The RCP used the relative risk reductions in readmissions seen in the Ottawa model to estimate that the NHS could release over GBP 60 million in healthcare costs and utilisation [28]. Furthermore, the OMSC and the RCP report were pivotal factors in the decision to commit major funding to hospital-based tobacco dependency treatment in the NHS long term plan.

Following the principles of the OMSC, a randomised controlled trial (RCT) set on the acute medical wards in a large teaching hospital in the UK in 2010/2011 investigated the effectiveness of the systematic identification of smoking status and opt out provision of cessation support relative to usual care [37]. The intervention comprised the delivery of bedside brief advice to quit, daily behavioural support, and cessation pharmacotherapy for the duration of the hospital admission for all smokers and recent ex-smokers (smoked within the last 4-weeks), with follow up and referral to community cessation services after discharge. In comparison, usual care comprised the ascertainment of smoking status on admission, in line with standard hospital practice, and cessation support delivered at the discretion of clinical staff. The study found that with delivery of a comprehensive opt-out stop smoking intervention at the bedside, 4-week and 6-month abstinence doubled in the intervention group, with a significant increase in uptake of inpatient behavioural support and use of pharmacotherapy. Qualitative evaluation of the study revealed that the service was deemed appropriate by both patients and healthcare providers, particularly the initiation of cessation support by specialist staff during an inpatient stay [38].

## 5. The CURE Project

Greater Manchester, a region in the North-West of England, has implemented a vanguard programme of hospital-based tobacco-dependency treatment services. The Greater Manchester CURE project (Conversation-Understand-Replace-Experts/Evidence-base) aimed to translate the OMSC and RCT evidence into a resilient, sustainable, and scalable real-world clinical service model. The service consisted of three key components. First, the service aimed to change the culture across the hospital system and place the key responsibility for the initial management of tobacco dependency in the hands of the admitting clinical team. The word ‘CURE’ was chosen specifically to medicalise tobacco dependency and focus the clinical teams on disease management. Specific CURE e-learning modules were developed to educate staff in the pathology of tobacco dependency, delivery of very brief advice, assessment of level of dependency, and to present a standardised prescribing protocol for patients that smoke admitted to the hospital. Very brief advice (VBA) is a short healthcare professional-delivered intervention that focuses on the offer of help, rather an instruction to stop smoking, and centred on three components: ask, advice, and act. During VBA, the healthcare professional identifies that a person smokes, advises that the best chance of stopping smoking is with the help of medications and specialist support, and then provides access to the medication and support. The prescribing protocol provides combination Nicotine Replacement Therapy (NRT) mapped to the level of dependency and recommends all patients that smoke are offered varenicline in conjunction with the NRT. In addition, standardised teaching sessions covering the same areas were delivered across the hospital during the launch, to support the e-learning package. The CURE e-learning modules were uploaded to the Hospital’s employee training platform (the learning hub) and were recommended to all staff to complete. The e-learning modules covered the background knowledge and pathology of tobacco dependency, how to deliver VBA, the standardised prescribing protocol, and provided video examples of consultations in different hospital settings between healthcare professional and patients that smoke, illustrating these interventions. Over one thousand staff members completed the training during the pilot. The e-learning modules were also made available to any healthcare professional via The CURE website, which provided certification on completion (https://thecureproject.co.uk/training/, accessed on 12 April 2022). The electronic patient record system was designed with new functionality to deliver a specific workflow, where smoking status was recorded for all patients on admission. If a patient was identified as a current smoker, the system provided prompts to deliver VBA and pharmacotherapy and record the completion of this. It also delivered an automated referral to the CURE team (opt-out referral pathway). Following the delivery of the admission bundle of VBA and pharmacotherapy by the admitting team, the CURE team (a team of specialist pay band level six tobacco dependency nurses) will approach every smoker and offer a review of pharmacotherapy, specialist behaviour change support, and motivational interviewing. A key part of this specialist consultation is also offering and agreeing a treatment and support plan after discharge. The CURE team offered ongoing medications and support following discharge, through regular outpatient consultations (face to face or telephone) for 12 weeks.

A pilot implementation study of the CURE project was completed at a single hospital in Greater Manchester over a six-month period in 2018/2019 [39]. A total of 14,690 adult admissions were recorded in hospital’s electronic patient record (EPR) system (excluding paediatrics, maternity, and day case admissions). Smoking status was recorded in 92% (13,515/14,690) and 18% (2393/13,515) were recorded as active smokers. This is higher than the overall UK smoking prevalence of 15%. Overall, 96% (2308/2393) of all patients who smoke were provided with brief advice at the point of admission, 66% (1568/2393) were prescribed some form of stop smoking pharmacotherapy, and 61% (1450/2393) accepted specialist assessment from the CURE team from the opt-out referral process. After discharge, 1105 (46%), 1179 (49%), and 800 (33%) patients completed post-discharge follow-up with the hospital CURE team at 2 weeks, 4 weeks, and 12 weeks, respectively. Overall, 495 (21%) and 525 (22%) patients self-reported to be abstinent from tobacco at 4 weeks (of which 293 were chemically validated) and 12 weeks, respectively. A health economic analysis of the CURE pilot demonstrated an intervention cost of GBP 40.21 per patient who smokes admitted to the hospital, plus GBP 97.40 of additional costs post-discharge in those patients completing post-discharge support. The cost per quit rate across the entire pathway was GBP 475. The return on investment (ROI) analysis produced a gross financial ROI ratio of GBP 2.12 return per GBP 1 invested, with a payback period of 4 years. The cashable financial ROI ratio is GBP 1.06 return per GBP 1 invested with a payback period of 10 years. The public value ROI ratio is GBP 30.49 per GBP 1 invested. The cost per QALY for this programme is GBP 487 [40]. The CURE project has now been implemented across seven out of 10 hospitals across Greater Manchester, with the final three hospitals going live in 2022. The rates of uptake of pharmacotherapy, specialist support, follow-up, and abstinence are greater than, or equivalent to, those seen in the OMSC, the UK RCT, and a large study of hospital-based tobacco dependency treatment in South Carolina of over 40,000 hospital admissions [24], suggesting this real-life service is at least replicating, or enhancing, the benefits seen in these published outcomes.

## 6. Challenges of Implementation of CURE and National NHS Long Term Plan Tobacco Programme

**Figure 3:**
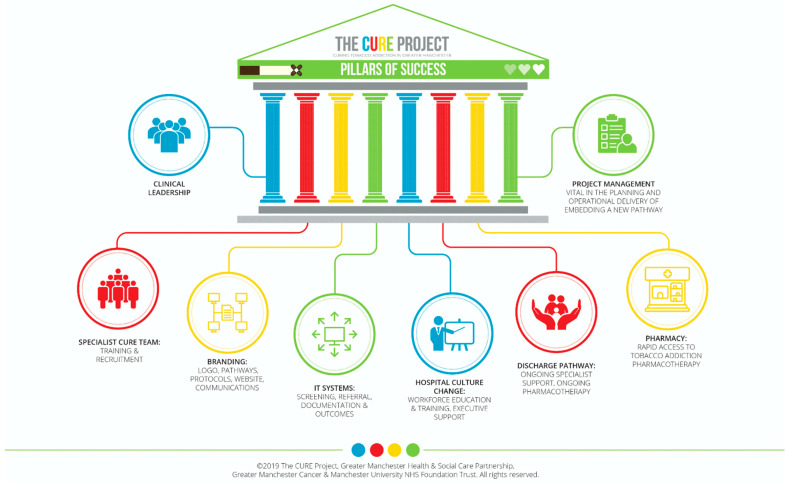
The pillars of success of the CURE Project.

**Figure 4:**
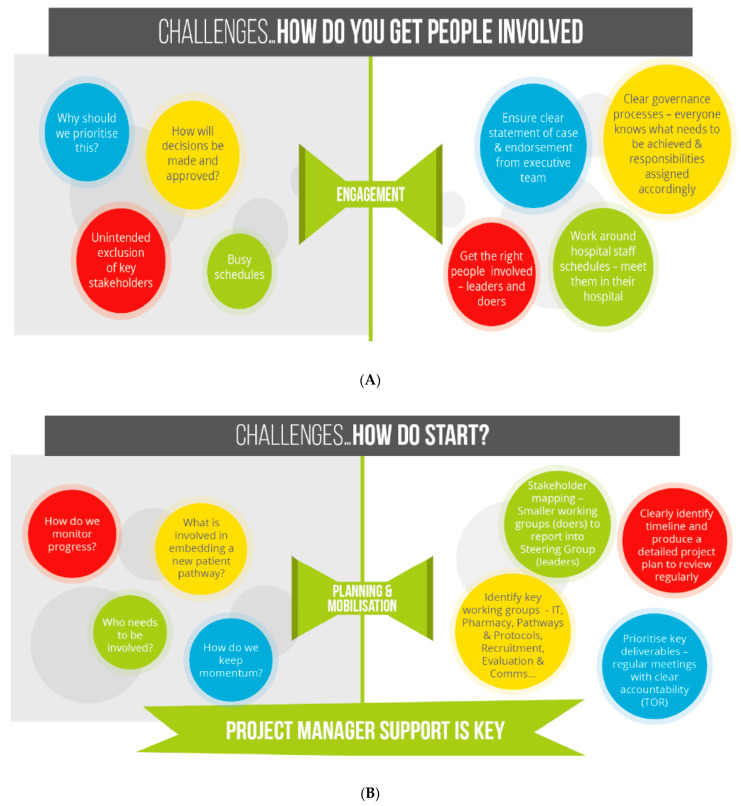

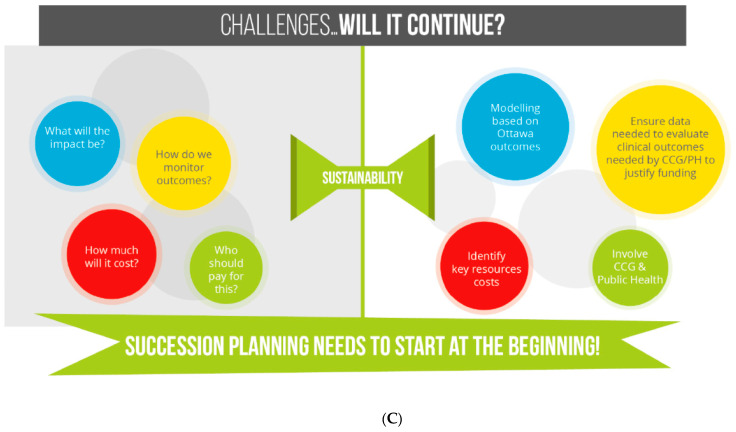
Operational and project management challenges& solutions during the implementation of the CURE Project: (**A**) How do you get people involved? (**B**) How do you start? (**C**) Will it continue?

The overarching challenge during the implementation of the CURE project was that it was delivering a new inpatient pathway that previously had not been considered a clinical pathway, but rather a lifestyle section of a patient’s medical history. This new service needed to fundamentally change the perception of smoking tobacco by medicalising tobacco dependency. What underpinned this was the need for a large-scale culture change and attitude towards smoking and smokers in general, not only by clinical staff, but the NHS as a whole, as well as patients and the general public accessing acute care. When reflecting on the implementation of this pathway, the CURE project team developed the “pillars of success” to capture what key components were required to deliver CURE at scale (Figure 3). The golden thread throughout was ensuring there was a strong communications and engagement strategy (before, after, and during implementation), from within an individual Trust, through to linking with regional and national campaigns to cover staff, patient, and public messaging. Developing a gold class service needs to be supported by promotion of its purpose and benefits, to maximise engagement and understanding. Medicalising the treatment of smokers and focusing on the change of terminology, from “lifestyle choice” to “dependency”, was a constant challenge and, therefore, this was always the focal point of all presentations. Specific e-learning training was developed for all staff, and educational resources were created for a wide range of clinical forums and groups, all supported openly from the top down. Ensuring a whole patient pathway approach in terms of planning, funding, and development with clinical and operational input also needs to be considered from the very beginning as a primary care engagement in an acute care-based service can be challenging if relationships are not developed between acute clinicians and GPs, to understand how integrating treatment for tobacco dependency needs to happen across all healthcare services to work best for patients. This was particularly pertinent during the up-scaling of the CURE project across numerous hospital sites, where the handover of care to community stop smoking services became a key aspect of post-discharge support. A further barrier to rollout and expansion was the desire to understand the potential cost and impact of treating all smokers admitted to hospital, and where this would and should be funded from, given the expectation of continued treatment after discharged from acute care. We, therefore, ensured that evaluation was at the forefront of the implementation process, from updating digital systems to capture new service data, through to the completion of a full cost–benefit analysis of a patient on a CURE pathway within a CCG that did not fund any commissioned community services, which enabled a full pathway review. Understanding what all key stakeholders require, in order to make the case for continued funding and support, from trust executives to public health colleagues, and including a robust evaluation plan that covers every aspect is important and integral to successful transformation programme management. A summary of specific operational and project management challenges, as well as solutions, are presented in Figure 4.

## 7. Conclusions

The NHS long term plan is a pivotal intervention to address the significant reduction of funding in tobacco dependency treatment in the UK, and an important strategy to address the unique harms that smoking tobacco causes to the population. This signals a renewed investment within the NHS to deliver tobacco dependency treatment within acute care settings and the opportunity to upskill and empower the medical workforce in the management of this deadly disease. The evidence base for effectiveness is substantial and the CURE project has provided a framework for real-world delivery, with significant clinical and cost effectiveness. There are challenges and barriers to overcome to achieve large-scale culture change and stakeholder engagement across a complex system of tobacco control, which provide lessons for implementation sites across the UK and beyond.

## Data Availability

The data presented in this study are available on request from the corresponding author.

## References

[1] Health AoSa ASH at 50: Nearly 8 Million Lives in the UK Lost Due to Tobacco since 1971. https://ash.org.uk/media-and-news/press-releases-media-and-news/ash-at-50-nearly-8-million-lives-in-the-uk-lost-due-to-tobacco-since-1971/.

[2] Anthenelli R.M., Benowitz N.L., West R., St Aubin L., McRae T., Lawrence D., Ascher J., Russ C., Krishen A., Evins A.E. (2016). Neuropsychiatric safety and efficacy of varenicline, bupropion, and nicotine patch in smokers with and without psychiatric disorders (EAGLES): A double-blind, randomised, placebo-controlled clinical trial. Lancet.

[3] Rigotti N.A., Clair C., Munafo M.R., Stead L.F. (2012). Interventions for smoking cessation in hospitalised patients. Cochrane Database Syst Rev..

[4] Excellence NIfHaC (2007). Varenicline for Smoking Cessation Technology Appraisal Guidance [TA123].

[5] Windle S.B., Filion K.B., Mancini J.G., Adye-White L., Joseph L., Gore G.C., Habib B., Grad R., Pilote L., Eisenberg M.J. (2016). Combination Therapies for Smoking Cessation: A Hierarchical Bayesian Meta-Analysis. Am. J. Prev. Med..

[6] Hajek P., Phillips-Waller A., Przulj D., Pesola F., Myers Smith K., Bisal N., Li J., Parrott S., Sasieni P., Dawkins L. (2019). A Randomized Trial of E-Cigarettes versus Nicotine-Replacement Therapy. N. Engl. J. Med..

[7] Hartmann-Boyce J.M.H., Lindson N., Bullen C., Begh R., Theodoulou A., Notley C., Rigotti N.A., Turner T., Butler A.R., Hajek P. (2020). Can Electronic Cigarettes Help People Stop Smoking, and Do They Have Any Unwanted Effects When Used for This Purpose?.

[8] UK Government The NHS Constitution. https://www.gov.uk/government/publications/the-nhs-constitution-for-england/the-nhs-constitution-for-england.

[9] Royal College of Physicians Smoking and Health. A Report on Smoking in Relation to Lung Cancer and Other Diseases 1962 27/06/2017. https://www.rcplondon.ac.uk/projects/outputs/smoking-and-health-1962.

[10] Department of Health (1998). Smoking Kills. A White Paper on Tobacco.

[11] Raw M., McNeill A., West R. (1998). Smoking cessation guidelines for health professionals. A guide to effective smoking cessation interventions for the health care system. Thorax.

[12] Sieber S., Gerber V., Jandova V., Rossano A., Evison J.M., Perreten V. (2011). Evolution of multidrug-resistant Staphylococcus aureus infections in horses and colonized personnel in an equine clinic between 2005 and 2010. Microb. Drug Resist..

[13] Excellence NIfHaC What We Do, Our Role. https://www.nice.org.uk/about/what-we-do.

[14] National Institute for Health and Care Excellence (NICE) Smoking: Brief Interventions and Referrals (PH 1); 2006 26/10/2018. www.nice.org.uk/guidance/ph1.

[15] National Institute for Health and Care Excellence (NICE) Stop Smoking Services PH102008 26/02/2018. nice.org.uk/guidance/ph10.

[16] National Institute for Health and Care Excellence (2013). Smoking Cessation in Secondary Care: Acute, Maternity and Mental Health Services.

[17] National Institute for Health and Care Excellence (NICE) Smoking: Stopping in Pregnancy and after Childbirth (PH26)2010 26/02/2018. www.nice.org.uk/guidance/ph26.

[18] National Institute for Health and Care Excellence (NICE) Stop Smoking Interventions and Services 2018 30/03/2018. https://www.nice.org.uk/guidance/ng92.

[19] National Institute for Health and Care Excellence Tobacco: Preventing Uptake, Promoting Quitting and Treating Dependence (NG209). https://www.nice.org.uk/guidance/ng209.

[20] The National Archive Health and Social Care Act 201215/02/2018. http://www.legislation.gov.uk/ukpga/2012/7/contents/enacted.

[21] UK Government Public Health Grant 2022 to 2023. https://www.gov.uk/government/publications/public-health-grants-to-local-authorities-2022-to-2023.

[22] The Health Foundation Why Greater Investment in the Public Health Grant Should Be a Priority. https://www.health.org.uk/news-and-comment/charts-and-infographics/why-greater-investment-in-the-public-health-grant-should-be-a-priority.

[23] NHS Digital Statistics on NHS Stop Smoking Services in England. https://digital.nhs.uk/data-and-information/publications/statistical/statistics-on-nhs-stop-smoking-services-in-england/april-2020-to-march-2021/datasets.

[24] Royal College of Physicians (London) Smoking and Health 2021: A Coming of Age for Tobacco Control?. https://www.rcplondon.ac.uk/projects/outputs/smoking-and-health-2021-coming-age-tobacco-control.

[25] British Thoracic Society (BTS) SA, Zaheer Mangera Smoking Cessation Audit Report Smoking Cessation Policy and Practice in NHS Hospitals, National Audit Period: 1 April–31 May 2016. https://www.brit-thoracic.org.uk/document-library/audit-and-quality-improvement/audit-reports/bts-smoking-cessation-audit-report-2016/.

[26] Davies N., Cheeseman H., Arnott D., Pierce E., Langley T.E., Murray R., Bogdanovica I., Bains M. (2022). When is subnational, supra-local tobacco control ‘just right’? A qualitative study in England. Nicotine Tob. Res..

[27] Beard E., West R., Michie S., Brown J. (2016). Association between electronic cigarette use and changes in quit attempts, success of quit attempts, use of smoking cessation pharmacotherapy, and use of stop smoking services in England: Time series analysis of population trends. BMJ.

[28] Tobacco Advisory Group of the Royal College of Physicians Hiding in Plain Sight: Treating Tobacco Dependency in the NHS2018 31/07/2018. https://www.rcplondon.ac.uk/file/10116/download?token=K05kvT-7.

[29] National Health Service The NHS Long Term Plan 2019 07/01/2019. https://www.longtermplan.nhs.uk/wp-content/uploads/2019/01/nhs-long-term-plan.pdf.

[30] Health Do Towards a Smokefree Generation: A Tobacco Control Plan for England. https://www.gov.uk/government/publications/towards-a-smoke-free-generation-tobaccocontrol-plan-for-england.

[31] Thorlby R.G.T., Everest G., Allen L., Shembavnekar N., Fisher R. The NHS Long Term Plan and COVID-19: Assessing Progress and the Pandemic’s Impact. https://www.health.org.uk/publications/reports/the-nhs-long-term-plan-and-covid-19.

[32] Mangera Z.D.N. (2020). British Thoracic Society National Smoking Cessation Audit Report 2019.

[33] Society B.T. Tobacco Dependency Project. https://www.respiratoryfutures.org.uk/programmes/tobacco-dependency-project/tobacco-dependency-project-resources/.

[34] Reid R.D., Mullen K.A., Slovinec D’Angelo M.E., Aitken D.A., Papadakis S., Haley P.M., McLaughlin C.A., Pipe A.L. (2010). Smoking cessation for hospitalized smokers: An evaluation of the "Ottawa Model". Nicotine Tob. Res..

[35] Reid R.D., Pipe A.L., Quinlan B. (2006). Promoting smoking cessation during hospitalization for coronary artery disease. Can. J. Cardiol..

[36] Mullen K.A., Manuel D.G., Hawken S.J., Pipe A.L., Coyle D., Hobler L.A., Younger J., Wells G.A., Reid R.D. (2017). Effectiveness of a hospital-initiated smoking cessation programme: 2-year health and healthcare outcomes. Tob. Control..

[37] Murray R.L., Leonardi-Bee J., Marsh J., Jayes L., Li J., Parrott S., Britton J. (2013). Systematic identification and treatment of smokers by hospital based cessation practitioners in a secondary care setting: Cluster randomised controlled trial. BMJ.

[38] Bains M., Britton J., Marsh J., Jayes L., Murray R.L. (2014). Patients‐ and healthcare professionals‐ views on a specialist smoking cessation service delivered in a United Kingdom hospital: A qualitative study. Tob. Induc. Dis..

[39] Evison M., Pearse C., Howle F., Baugh M., Huddart H., Ashton E., Rutherford M., Kearney C., Elsey L., Staniforth D. (2020). Feasibility, uptake and impact of a hospital-wide tobacco addiction treatment pathway: Results from the CURE project pilot. Clin. Med..

[40] Evison M., Cox J., Howle F., Groom K., Moore R., Clegg H., Pearse C., Rutherford M., Tempowski A., Grundy S. (2021). Health economic analysis for the ‘CURE Project’ pilot: A hospital-based tobacco dependency treatment service in Greater Manchester. BMJ Open Respir. Res..

